# Combining WGCNA and machine learning to identify mechanisms and biomarkers of hyperthyroidism and atrial fibrillation

**DOI:** 10.3389/fcvm.2025.1694255

**Published:** 2025-11-20

**Authors:** Linyuan Wang, Kun Yang, Ruilong Kang, Pengbo Liu, Yongzhi Deng

**Affiliations:** 1 Department of Cardiovascular Surgery, The Affiliated Hospital of Shanxi Medical University, Shanxi Cardiovascular Hospital (Institute), Shanxi Clinical Medical Research Center for Cardiovascular Disease, Taiyuan, China; 2 Department of Cardiovascular Surgery, Shanxi Cardiovascular Hospital (Institute), the Affiliated Hospital of Shanxi Medical University, Shanxi Clinical Medical Research Center for Cardiovascular Disease, Taiyuan, China

**Keywords:** hyperthyroidism, atrial fibrillation, weighted gene co-expression network analysis, machine learning, biomarkers

## Abstract

**Background:**

Hyperthyroidism and atrial fibrillation (AF) are interrelated conditions with significant cardiovascular impact. While their clinical association is established, the molecular mechanisms remain unclear. Identifying shared biomarkers and pathways can advance understanding and guide therapy.

**Methods:**

The hyperthyroidism dataset GSE71956 and the AF dataset GSE115574 were obtained from the Gene Expression Omnibus (GEO) database. Differential gene analysis was performed using the “limma” package, and overlapping genes shared by both diseases were identified through weighted gene co-expression network analysis (WGCNA), followed by functional enrichment analysis. Machine learning algorithms were also applied to identify key biomarkers. To validate the predictive results, peripheral blood samples were collected for real-time quantitative polymerase chain reaction (RT-qPCR) analysis. Finally, immune infiltration analysis was conducted to evaluate immune cell changes in hyperthyroidism and AF.

**Results:**

Through differential gene screening and WGCNA, 23 overlapping genes associated with hyperthyroidism and AF were identified. Using least absolute shrinkage and selection operator (LASSO) and random forest (RF) machine learning algorithms, CXCL16 and TMEM127 were ultimately identified as key genes. The two genes demonstrated good diagnostic efficacy in the hyperthyroidism validation set GSE276271 (AUC: TMEM127, 0.636; CXCL16, 0.591) and in the AF validation set GSE2240 (AUC: TMEM127, 0.745; CXCL16, 0.720). RT–qPCR analysis demonstrated that CXCL16 and TMEM127 expression levels were significantly elevated in both the hyperthyroidism and AF groups compared to the control group, aligning with the findings from our prior bioinformatics analysis. Immune analysis revealed significant differences in two immune cell types in both hyperthyroidism and AF.

**Conclusion:**

CXCL16 and TMEM127 are promising biomarkers, offering insights into the shared pathogenesis of hyperthyroidism and AF. These findings provide a foundation for novel diagnostic and therapeutic strategies targeting these conditions.

## 1 Introduction

Hyperthyroidism, a prevalent endocrine disorder, is particularly common in iodine-deficient regions, with a global prevalence of 0.2%–1.3% in iodine-sufficient populations (1). The condition is characterized by elevated levels of thyroxine (T4), triiodothyronine (T3), or both, significantly impacting cardiac energy metabolism, cardiovascular function, and the heart's electrical conduction system (2). Cardiovascular complications such as sinus tachycardia and atrial fibrillation (AF) are commonly associated with hyperthyroidism.

AF, the most widespread sustained arrhythmia globally, has become a critical public health issue due to its increasing incidence, associated healthcare burdens, and adverse effects on morbidity and mortality (3). Major risk factors for AF include aging, sedentary lifestyles, obesity, diabetes, metabolic syndrome, and obstructive sleep apnea (4, 5). Moreover, genetic predisposition plays a significant role in AF, with over 140 genetic loci identified as contributors to its pathogenesis (6, 7).

The connection between hyperthyroidism and AF has been acknowledged for over a century (8). For example, a population-based study involving more than 40,000 individuals with hyperthyroidism revealed that 8.3% experienced AF or atrial flutter within a month of diagnosis (9). Recent machine learning analyses of extensive datasets have further substantiated this relationship (10). Additionally, studies from Denmark have indicated a heightened risk of hyperthyroidism following AF onset, emphasizing the importance of thyroid function monitoring after an AF diagnosis (11). Despite these advances, the molecular mechanisms underpinning this relationship remain poorly understood, warranting further research into potential therapeutic strategies.

The advent of high-throughput technologies and bioinformatics has revolutionized the discovery of biomarkers and therapeutic targets. While traditional bioinformatics research largely focused on differential gene expression and protein-protein interaction (PPI) network analyses, these methods often overlooked co-expressed gene clusters shared by hyperthyroidism and AF (12, 13). Furthermore, the precision of PPI network analyses has been questioned. Newer approaches, such as weighted gene co-expression network analysis (WGCNA) and machine learning (ML) algorithms, have emerged as powerful tools for identifying disease-relevant targets. WGCNA identifies disease-associated gene modules by constructing scale-free networks, while machine learning algorithms like Least Absolute Shrinkage and Selection Operator (LASSO) and Random Forest (RF) are increasingly utilized to detect biomarkers and enhance diagnostic accuracy (14).

**Figure 1:**
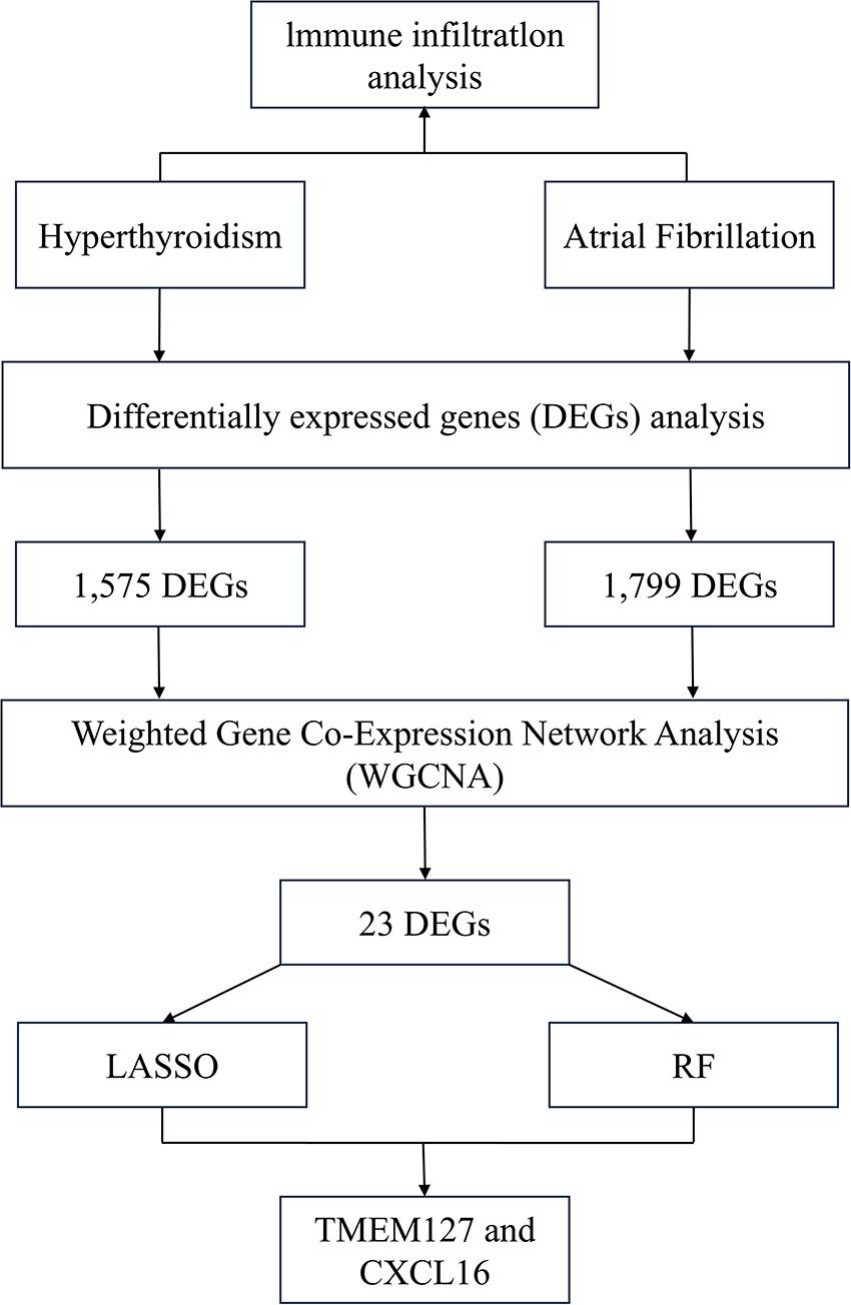
Flowchart of the study.

This study employed mRNA expression datasets from the GEO database to identify co-expression modules shared by hyperthyroidism and AF using WGCNA. Functional enrichment analysis was conducted to explore the biological roles of overlapping genes, while LASSO and RF approaches were applied to pinpoint potential biomarkers. These biomarkers were validated using an independent dataset. To further validate our findings, peripheral blood samples were collected for real-time quantitative polymerase chain reaction (RT-qPCR) analysis. Additionally, immune cell infiltration analysis was performed to investigate immune cell involvement in the pathogenesis of hyperthyroidism and AF. Figure 1 depicts the study flowchart.

## 2 Materials and methods

### 2.1 Data collection

Gene expression profiles of hyperthyroidism and AF were obtained from the GEO database (https://www.ncbi.nlm.nih.gov/geo). The GSE115574 dataset (microarray data on the GPL570 platform) includes 59 atrial tissue samples from 30 patients who underwent mitral regurgitation repair surgery, comprising 28 samples from individuals with AF and 31 from those with sinus rhythm (SR). The GSE71956 dataset (microarray data on the GPL10558 platform) contains 49 peripheral blood CD4^+^ T lymphocyte samples, including 31 from patients with hyperthyroidism and 18 from healthy controls. To further validate our findings, we selected datasets GSE2240 and GSE276271 as independent validation sets. The GSE2240 dataset (microarray data on the GPL97 platform) includes right atrial tissue samples from 30 patients who underwent cardiac surgery (either valve repair or coronary artery bypass grafting), with 10 samples from patients with AF and 20 from individuals with SR. The GSE276271 dataset (RNA-seq data on the GPL28702 platform) comprises 15 feline thyroid tissue samples, including 11 from hyperthyroidism models and 4 from healthy controls. All the datasets were subjected to standardized data preprocessing.

### 2.2 Identification of differentially expressed genes (DEGs)

DEGs were analyzed using the “limma” R package by comparing hyperthyroidism samples against controls in the GSE71956 dataset and AF samples against controls in the GSE115574 dataset. Heatmaps and volcano plots were generated with the “pheatmap” and “ggplot2” R packages. Genes with |log2FC| > 0.1 and *p*-value < 0.05 were deemed significant.

### 2.3 Weighted gene co-expression network analysis

A co-expression network was constructed using the “WGCNA” R package, with DEGs as input. Hierarchical clustering identified and excluded outliers. A scale-free network was created using the “pickSoftThreshold” function to determine the optimal soft threshold power (*β*), transforming the similarity matrix into a weighted adjacency matrix. From this, a topological overlap matrix (TOM) was derived to enhance noise reduction. Gene modules were identified through hierarchical clustering and the dynamic tree cut algorithm. Pearson correlation analysis was used to correlate gene modules with clinical traits, and modules showing strong correlations were visualized in a trait-gene network. Shared key genes were identified by intersecting hyperthyroidism- and AF-associated modules.

### 2.4 Functional enrichment analysis

Key genes were functionally annotated using Gene Ontology (GO) analysis, categorizing them into biological processes (BP), cellular component (CC), and molecular function (MF). KEGG pathway analysis identified the pathways and biological roles associated with these genes (15–17).

### 2.5 Machine learning

To identify robust diagnostic biomarkers, we applied LASSO regression and Random Forest (RF), with hyperparameters optimized to mitigate overfitting. For LASSO (R package “glmnet”), the optimal regularization parameter (*λ*) was determined by ten-fold cross-validation, selecting the *λ* value that minimized the cross-validated error. Genes with non-zero coefficients at this *λ* were retained. For RF (R package “randomForest”), the model was first run with 500 trees, and the optimal number of trees was identified as the point where the out-of-bag (OOB) error reached a minimum. A final model was built using this optimal number. From this model, the top 15 genes were selected based on the mean decrease in importance. Biomarkers were determined by intersecting genes identified by both algorithms. Receiver operating characteristic (ROC) analysis in the GSE276271 and GSE2240 datasets validated the diagnostic performance of the identified biomarkers.

### 2.6 Study population and blood samples

Peripheral blood samples were collected from 16 patients with hyperthyroidism and 16 patients with AF at the Cardiovascular Hospital affiliated with Shanxi Medical University. Inclusion criteria for the hyperthyroidism group were: (1) a confirmed clinical diagnosis of hyperthyroidism (18); (2) age ≥18 years. Exclusion criteria included: (1) concomitant AF or other arrhythmias; (2) the presence of autoimmune diseases, active infections, coagulation disorders, malignancies, psychiatric disorders, or neurological conditions. For the AF group, inclusion criteria were: (1) a confirmed diagnosis of AF (19); (2) age ≥18 years. Exclusion criteria included: (1) concurrent hyperthyroidism; (2) other arrhythmias requiring clinical intervention, as well as autoimmune diseases, active infections, coagulation disorders, malignancies, psychiatric disorders, or neurological conditions. Additionally, 16 healthy individuals undergoing routine physical examinations at the same hospital during the same period were recruited as the control group. These individuals had no history of endocrine or cardiovascular disease, and were matched with the study groups for age and sex. The study was approved by the hospital's ethics committee (2025WJ025), and informed consent was obtained from all participants or their legal guardians.

### 2.7 Real-time quantitative PCR

Total RNA was extracted from peripheral blood samples using an RNA extraction kit (Seven Biotech, Beijing, China), and stored at −80 °C. The RNA was then reverse-transcribed into complementary DNA (cDNA) using a reverse transcription kit from the same manufacturer. RT-qPCR was subsequently performed on a real-time PCR detection system using SYBR Green PCR Master Mix (Seven Biotech, Beijing, China), following the manufacturer's protocol. The expression levels of CXCL16 and TMEM127 were normalized to GAPDH as the internal control, and relative gene expression was calculated using the 2^−ΔΔCT^ method. Primer sequences used for RT-qPCR are listed in Supplementary Table S1.

### 2.8 Immune infiltration analysis

Immune infiltration was analyzed using CIBERSORT to estimate the proportions of immune cell types based on gene expression data. Immune cell profiles were compared between disease and control groups for both hyperthyroidism and AF datasets.

### 2.9 Statistical analysis

Statistical analyses were performed using GraphPad Prism 9.0 (GraphPad Software, La Jolla, California) and R (version 4.4.1). All data are presented as mean ± SEM. Statistical significance between two groups was analyzed using an unpaired Student's *t*-test. When more than two groups were involved, one-way ANOVA was used to analyze differences between groups. A *p*-value less than 0.05 was considered statistically significant.

## 3 Result

### 3.1 Identification of DEGs

**Figure 2:**
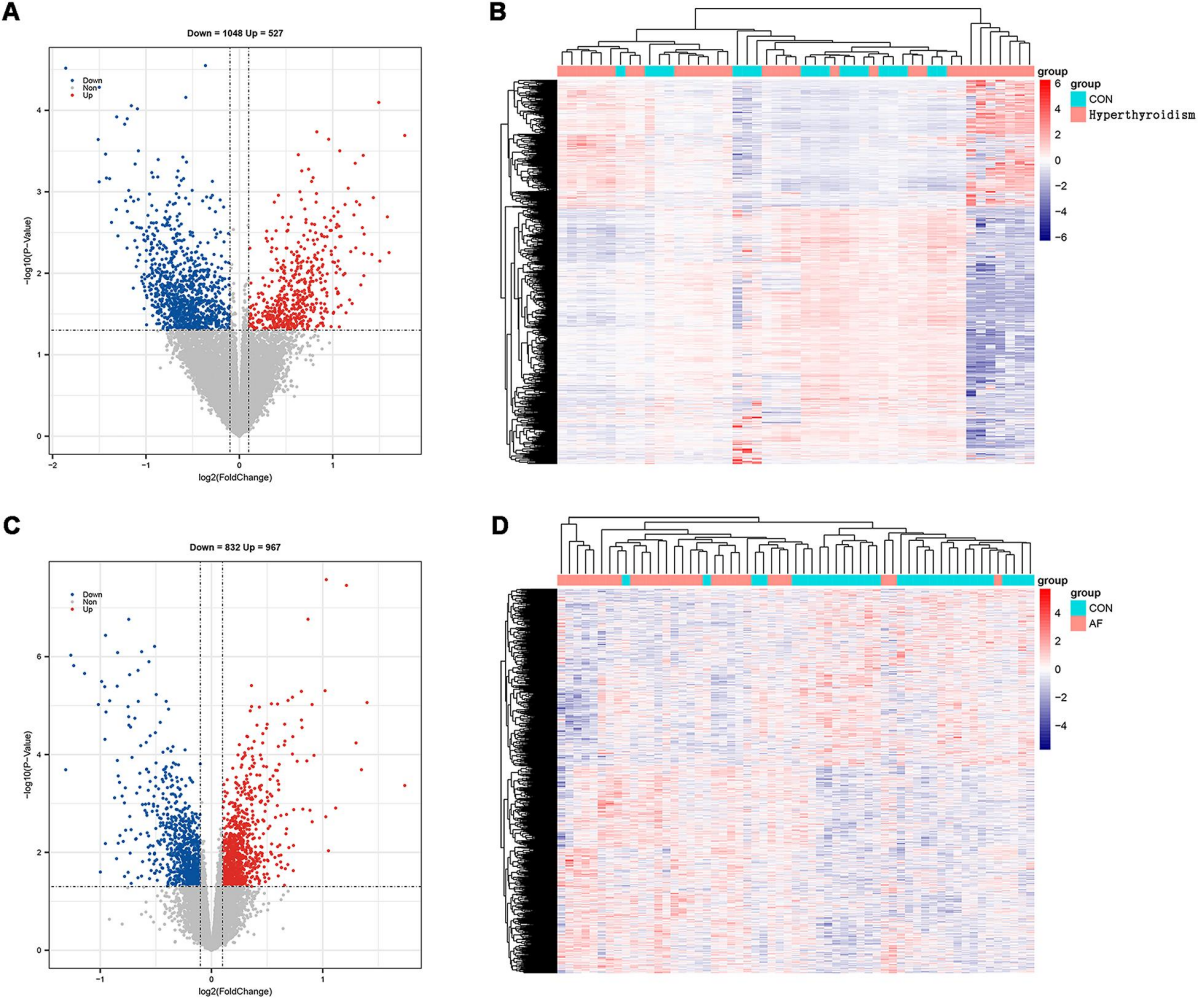
Screening of DEGs. **(A)** Volcano map of hyperthyroidism group; **(B)** heatmap of hyperthyroidism group; **(C)** volcano map of AF group; **(D)** heatmap of AF group.

Using R software for data analysis, we identified 1,575 DEGs in the hyperthyroidism dataset (GSE71956), comprising 527 upregulated and 1,048 downregulated genes (Figures 2A,B). In the AF dataset (GSE115574), a total of 1,799 DEGs were identified, with 967 upregulated and 832 downregulated genes (Figures 2C,D). The findings are summarized in Supplementary Table S2.

### 3.2 Weighted gene co-expression network analysis

**Figure 3:**
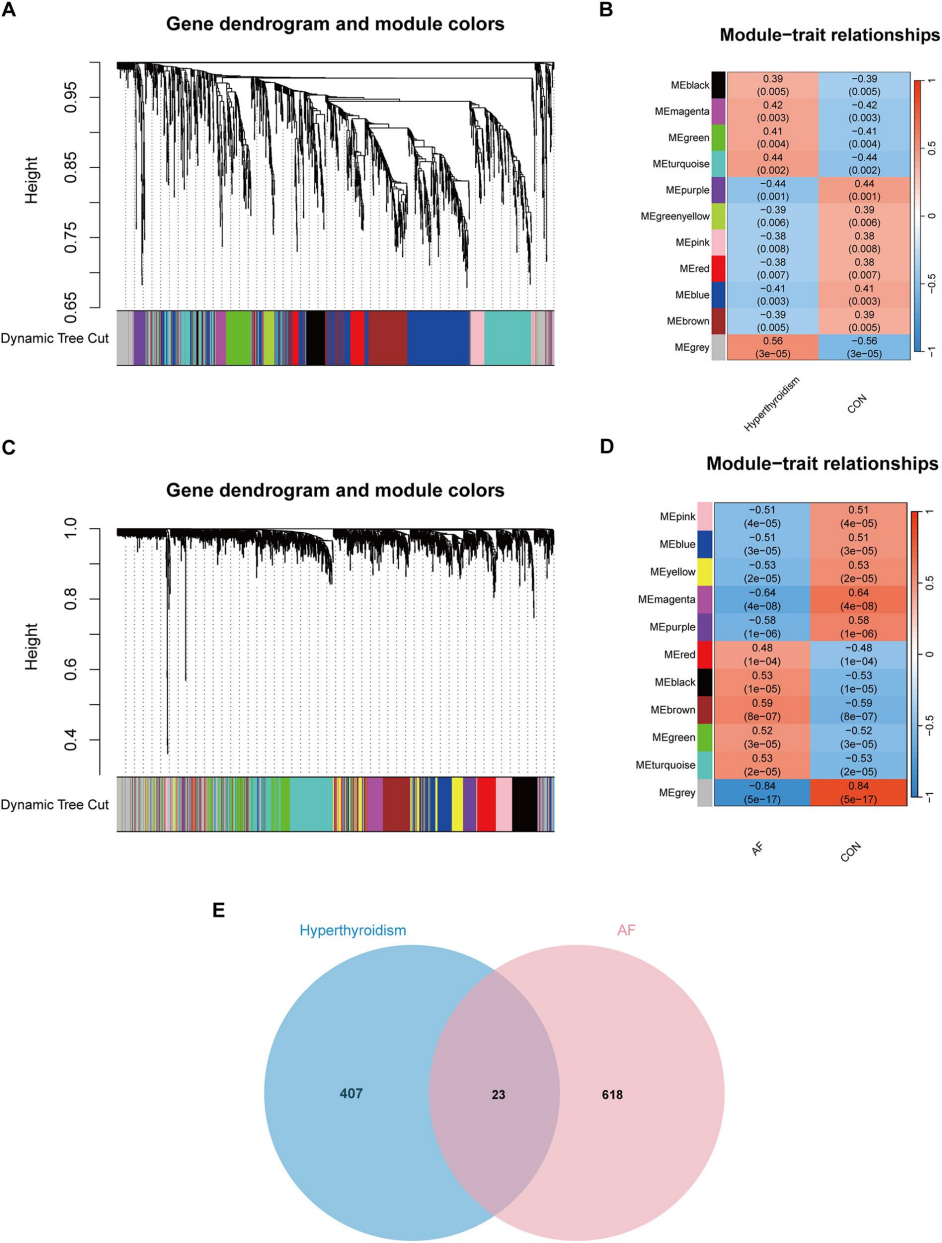
WGCNA analysis result. **(A)** Dendrograms for gene and trait clustering in hyperthyroidism were created. These gene clustering trees, or dendrograms, were derived from hierarchical clustering based on neighbor-related differences. **(B)** The hyperthyroidism condition was characterized by 11 gene co-expression modules. Each cell within these modules displays the correlation coefficient and the corresponding *p*-value. **(C)** Dendrograms for gene and trait clustering were also constructed for AF. **(D)** 11 gene co-expression modules of AF. **(E)** The intersection of hyperthyroidism and AF. We intersected the results to get 23 key genes.

WGCNA was performed on the DEGs from both the hyperthyroidism and AF datasets to identify modules significantly associated with these conditions. For the hyperthyroidism dataset, a soft thresholding power (*β*) of 12 was selected, resulting in a scale-free network. Eleven modules were identified, with the grey module (correlation coefficient = 0.56) and turquoise module (correlation coefficient = 0.44) showing the strongest positive associations with hyperthyroidism, collectively containing 430 genes. In the AF dataset, a scale-free network was achieved with *β* = 8. Eleven modules were identified, with the brown (correlation coefficient = 0.59), black (correlation coefficient = 0.53), and turquoise (correlation coefficient = 0.53) modules exhibiting the highest positive correlations with AF, including a total of 641 genes. By intersecting key modules from both datasets, 23 overlapping genes were identified as potential key genes (Figure 3). The key modules results are provided in Supplementary Table S3.

### 3.3 Functional enrichment analysis

**Figure 4:**
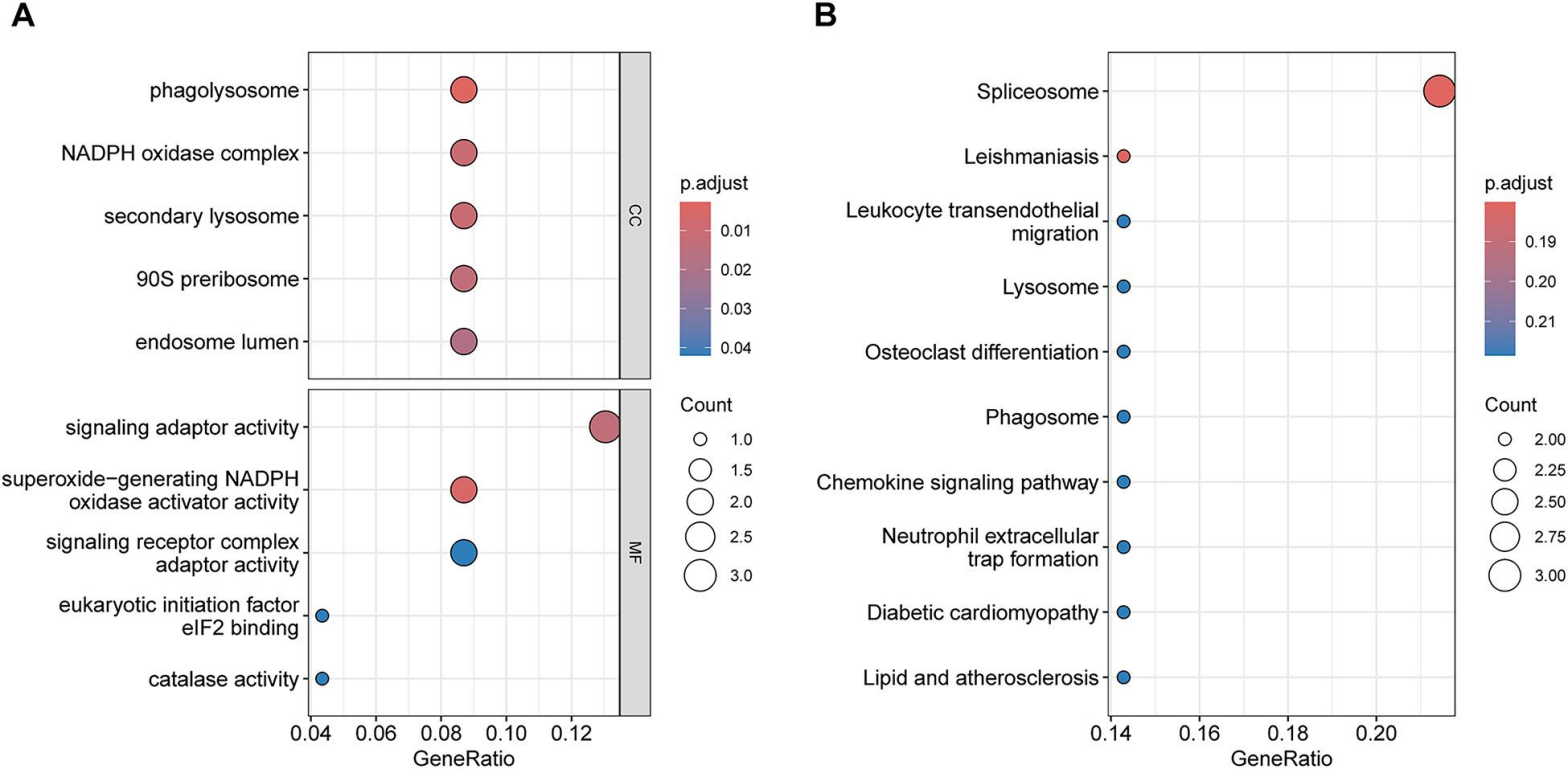
Functional enrichment analysis of Key genes. **(A)** GO enrichment analysis results. **(B)** KEGG enrichment analysis results.

GO and KEGG pathway enrichment analyses were conducted on the 23 key genes to explore shared biological processes underlying hyperthyroidism and AF. In the CC category, enriched terms included phagolysosome, NADPH oxidase complex, secondary lysosome, 90S preribosome, and endosome lumen. In the MF category, significant terms included superoxide-generating NADPH oxidase activator activity, signaling adaptor activity, signaling receptor complex adaptor activity, eukaryotic initiation factor eIF2 binding, and catalase activity. The top five enriched GO terms and the ten most enriched KEGG pathways are shown in Figure 4, highlighting the most relevant biological insights.

### 3.4 Machine learning screening for biomarkers

**Figure 5:**
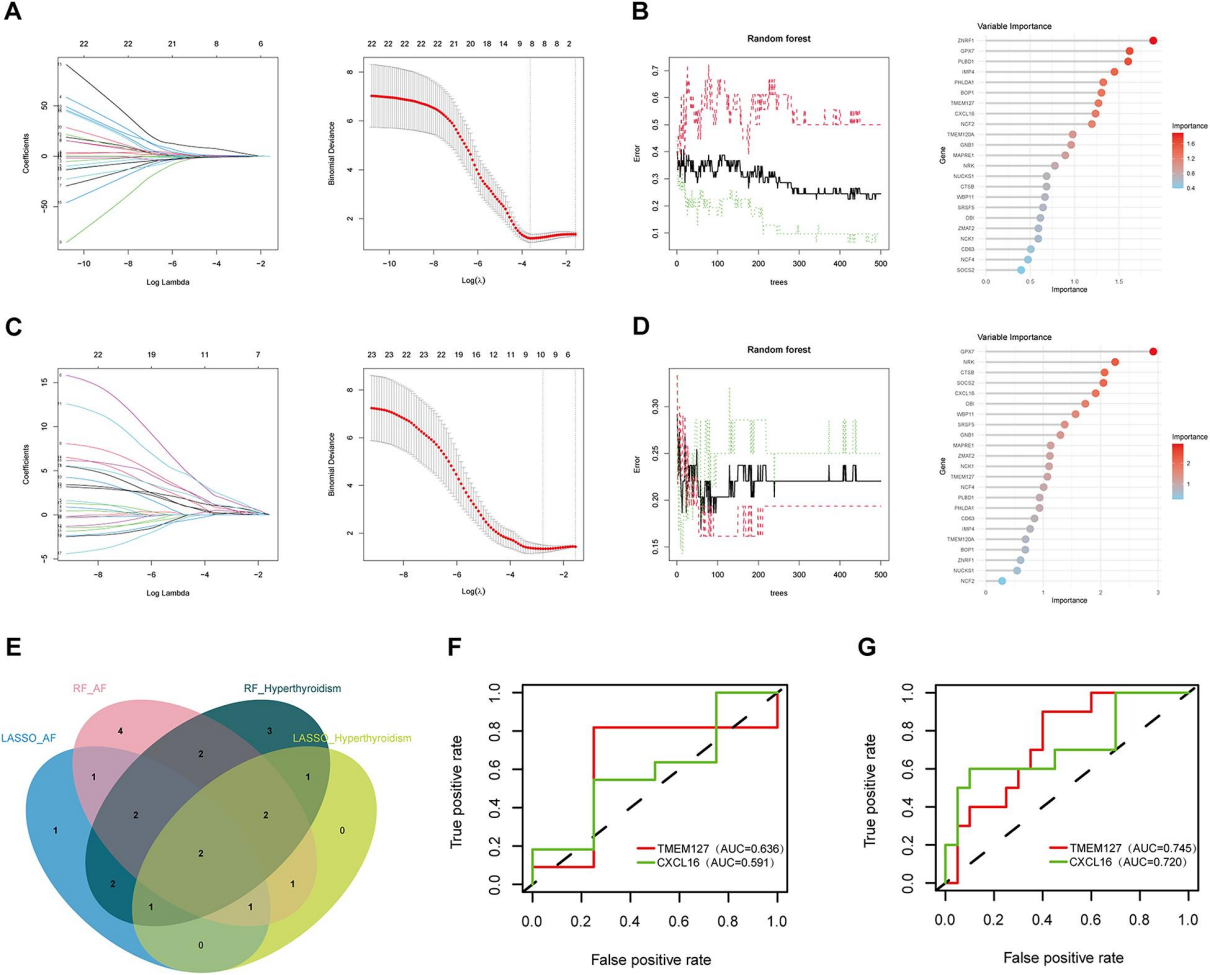
Biomarker screening and ROC validation. **(A)** LASSO regression model results in hyperthyroidism group; **(B)** RF results in hyperthyroidism group; **(C)** LASSO regression model results in AF group; **(D)** RF results in AF group; **(E)** Venn diagrams for Lasso and RF models; **(F)** the ROC of the biomarker genes in the hyperthyroidism validation set; **(G)** the ROC of the biomarker genes in the AF validation set.

Two ML algorithms, LASSO and RF, were employed to analyze the 23 candidate genes. LASSO regression identified eight significant genes associated with hyperthyroidism and ten with AF (Figures 5A,C). Concurrently, RF analysis ranked the top 15 most important genes for both conditions (Figures 5B,D). By intersecting the results from both algorithms, two key biomarker genes, CXCL16 and TMEM127, were identified (Figure 5E). ROC validation was conducted for two biomarker genes using the GSE276271 and GSE2240 validation datasets. In the hyperthyroidism validation dataset, CXCL16 and TMEM127 achieved area under the curve (AUC) values of 0.636 and 0.591, respectively, while in the AF validation dataset, the values were 0.745 and 0.720, demonstrating their diagnostic potential for both diseases (Figures 5F,G). All results of machine learning are presented in Supplementary Table S4.

### 3.5 Expression of diagnostic genes in clinical samples

**Figure 6:**
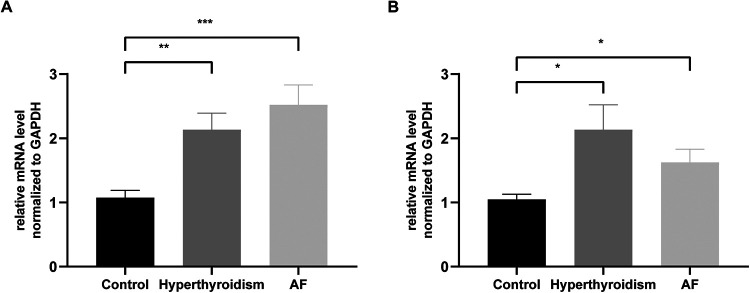
Validation of CXCL16 and TMEM127 expression in the hyperthyroidism and AF groups, as measured by RT-qPCR. **(A)** Relative expression of CXCL16. **(B)** Relative expression of TMEM127. Significance was indicated as *p < 0.05, **p < 0.01, ***p < 0.001.

To further validate our findings, a total of 48 clinical blood samples (16 patients with hyperthyroidism, 16 patients with AF, and 16 controls) from patients were collected. RT–qPCR analysis revealed that the expression levels of CXCL16 and TMEM127 were significantly elevated in both the hyperthyroidism and AF groups compared to the control group, corroborating the results of our bioinformatics analysis (Figure 6). Detailed clinical information for all patients is provided in Supplementary Table S5.

### 3.6 Immune infiltration analysis

**Figure 7:**
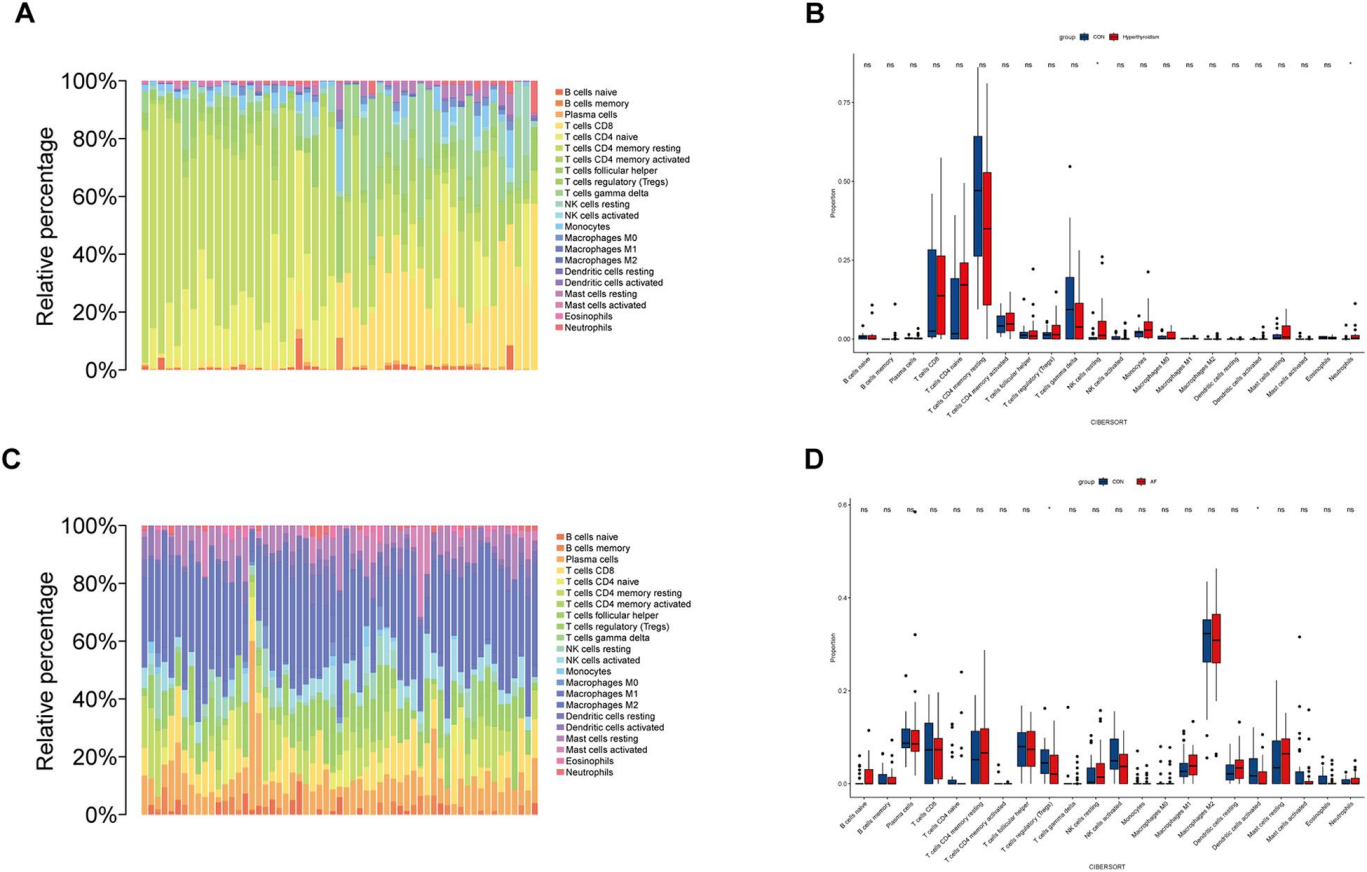
Immune cell infiltration analysis. **(A)** This bar plot illustrates the distribution of 22 distinct immune cell types across each sample in hyperthyroidism dataset, providing a comparative visual representation of their proportions. **(B)** The box plot depicts the expression profiles of 22 immune cell types in hyperthyroidism samples compared to control samples. **(C)** Similar to hyperthyroidism, the bar plot in AF dataset delineates the proportion of 22 immune cell types, offering a visual comparison of their distribution in different AF samples. **(D)** The box plot depicts the expression profiles of 22 immune cell types in AF samples compared to control samples. *p < 0.05.

To explore the role of immune cells in the pathogenesis of hyperthyroidism and AF, immune infiltration analysis was conducted using the CIBERSORT algorithm (Figures 7A,C). A comparison of immune cell composition between disease and control groups revealed significant differences. Hyperthyroidism samples exhibited elevated levels of resting natural killer (NK) cells and neutrophils (Figure 7B). In contrast, AF samples showed a reduced proportion of regulatory T cells (Tregs) and activated dendritic cells (Figure 7D).

## 4 Discussion

AF is a prevalent cardiac condition, affecting approximately 60 million people worldwide. It is the most common cardiac complication in hyperthyroidism, affecting 5%–15% of individuals with overt hyperthyroidism (8). Research from the United Kingdom indicates that patients with Graves' disease have more than twice the risk of developing AF compared to the general population (20). Additionally, AF in individuals with Graves' disease is associated with increased risks of acute coronary syndromes, stable angina, cardiac hospitalization, and overall mortality (21). Despite these associations, the mechanisms underlying the coexistence of hyperthyroidism and AF, as well as their common biomarkers, remain poorly understood, necessitating further investigation to identify specific and sensitive biomarkers.

WGCNA, a powerful bioinformatics tool for constructing gene co-expression networks from high-throughput microarray data, has gained popularity when combined with machine learning methods (22). Unlike traditional approaches, WGCNA establishes connections between gene expression and clinical data, enabling the discovery of novel therapeutic targets and providing insights into the shared pathogenesis of comorbidities (23). For example, Zhang et al. identified GLUL, NCF2, S100A12, and SRGN as significant biomarkers associated with AF and heart failure (HF), providing a foundation for clinical diagnosis and treatment (24). Similarly, WGCNA, integrated with differential gene and PPI network analyses, has been used to identify hub genes linked to cardioembolic stroke and AF, aiding in stroke diagnosis and prevention strategies (25). Other studies, such as that by Huang et al., identified STAT4 and COL1A2 as biomarkers for HF and depression comorbidities, broadening therapeutic possibilities (26). Zhu et al. further demonstrated the potential of combining WGCNA and ML to identify diagnostic biomarkers like NPPA, OMD, and PRELP for dilated cardiomyopathy with HF (27). These studies indicate that shared biomarkers may provide valuable insights into the complex interplay between hyperthyroidism and AF, thereby enhancing patient prognosis.

In this study, we retrieved gene expression data and clinical information from the GEO database to construct gene co-expression networks using WGCNA. Two machine learning algorithms, LASSO and RF, were employed to identify CXCL16 and TMEM127 as biomarkers significantly associated with both hyperthyroidism and AF. External validation confirmed the diagnostic utility of these two hub genes. To further validate our findings, we conducted RT-qPCR analyses on clinical blood samples. The results demonstrated that the gene expression levels of CXCL16 and TMEM127 were significantly upregulated in both the hyperthyroidism and AF groups compared to the control group. These findings are consistent with our previous bioinformatics analyses.

CXCL16 is a multifunctional CXC chemokine that primarily binds to the CXC chemokine receptor 6 (CXCR6), playing a critical role in immune regulation, inflammatory responses, and cell chemotaxis (28). In immune cell adhesion, CXCL16 facilitates the attachment of immune cells to endothelial and dendritic cells, contributing to the pathogenesis of multiple autoimmune diseases. Studies have demonstrated a strong association between CXCL16 levels and the severity, disease activity, and prognosis of conditions such as multiple sclerosis, autoimmune hepatitis, rheumatoid arthritis, Crohn's disease, and psoriasis (29). In cardiovascular research, serum CXCL16 levels have been positively correlated with the severity of coronary artery disease, highlighting its potential as a biomarker for cardiovascular risk assessment (30). Additionally, CXCL16 has been shown to promote Ly6Chigh monocyte infiltration, exacerbating cardiac dysfunction following acute myocardial infarction (31). Furthermore, elevated plasma CXCL16 levels have been associated with poor clinical outcomes and a higher recurrence rate in patients with AF (32). Although no direct studies have established a link between CXCL16 and hyperthyroidism, its crucial role in immune regulation and inflammation suggests potential involvement in immunopathological processes of hyperthyroidism.

TMEM127 is a transmembrane protein encoded by the TMEM127 gene and is widely expressed across various human tissues (33). As a tumor suppressor, germline mutations in TMEM127 are associated with hereditary pheochromocytomas and paragangliomas. These mutations typically result in a loss of TMEM127 function, leading to aberrant activation of the mTOR signaling pathway and promoting tumor development (34). Additionally, TMEM127 has been implicated in metabolic disorders such as insulin resistance and fatty liver disease, with studies demonstrating a strong correlation between its expression levels and insulin sensitivity (35). Notably, TMEM127 forms a ternary complex with SUSD6 and MHC-I, facilitating the recruitment of WWP2 to mediate the ubiquitination and lysosomal degradation of MHC-I. This mechanism enables cancer cells to downregulate MHC-I expression on their surface, thereby evading immune surveillance (36). Although a direct link between TMEM127 and AF or hyperthyroidism has not yet been established, its crucial role in key signaling pathways and cellular regulation suggests potential relevance. Further investigation into the roles of CXCL16 and TMEM127 in cardiovascular and endocrine metabolic disorders may provide novel insights and directions for future research.

Immune infiltration is a key factor in the progression of AF and hyperthyroidism, with immune cells playing a crucial role in mediating inflammation and other immune processes. Research indicates that abnormal thyroid hormone levels can impact the cardiovascular system by activating inflammatory pathways and oxidative stress responses, with dynamic immune cell changes central to this process (37). Specifically, patients with hyperthyroidism often exhibit a systemic inflammatory state, characterized by elevated levels of pro-inflammatory cytokines such as interleukin-6 (IL-6) and tumor necrosis factor-α (TNF-α). These cytokines contribute to thyroid tissue damage and dysfunction through multiple mechanisms (38), potentially linked to the abnormal activation of neutrophils and resting NK cells. In this study, hyperthyroid samples showed a significant increase in resting NK cells and neutrophils, with a similar trend observed in AF samples. This suggests that both conditions may share common immune-inflammatory activation mechanisms. In AF, the inflammatory response is marked by increased levels of IL-1β, TNF-α, and IL-6 (39). NK cells may contribute to AF-related inflammation by secreting these cytokines, promoting atrial fibrosis and structural remodeling. Neutrophils, which are typically absent in healthy cardiac tissue, respond rapidly to cardiac stress, peaking within 24 h—much earlier than inflammatory monocytes and lymphocytes. Upon activation, neutrophils adhere to and migrate toward injury sites, recruiting additional immune cells via chemokine concentration gradients (40). Furthermore, the interaction between neutrophils and endothelial cells triggers a respiratory burst, leading to oxidative damage to cardiomyocytes and myocardial tissue contraction (41). Tregs, a key subset of T cells involved in immune regulation, mitigate inflammation by inhibiting the activation of helper T cells (Th cells) and effector T cells, thereby reducing immune-mediated myocardial damage (42). Tregs are thought to play a critical immunomodulatory role in AF pathogenesis, and restoring their balance or enhancing their activity has been proposed as a therapeutic strategy to mitigate inflammation-driven atrial remodeling. This approach has been validated in atherosclerosis studies (43). Similarly, in Graves' disease, systemic inflammation is accompanied by a decline in Treg immunosuppressive function and a shift toward a cytotoxic phenotype (44). In addition, activated dendritic cells may contribute to the pathogenesis of both hyperthyroidism and AF through antigen presentation, inflammatory cytokine secretion, and interactions with other immune cells. Studies suggest that dendritic cells facilitate cardiac remodeling and functional recovery following myocardial infarction by modulating Tregs and macrophage polarization (45). However, in this study, immune cell infiltration patterns differed between hyperthyroidism and AF, potentially due to variations in myocardial vs. peripheral blood T cell samples. Despite their differences, growing evidence supports the central role of inflammation and immune responses in the onset, progression, and prognosis of both hyperthyroidism and AF. Further research into immune cell interactions and regulatory mechanisms may offer a more robust theoretical basis for optimizing clinical management and therapeutic strategies for these conditions.

However, this study has several limitations. Variability in sample sources may introduce bias, and datasets with sufficiently large sample sizes remain limited. Furthermore, it is unclear whether elevated mRNA levels correspond to proportional increases in protein expression, as many biological processes are also regulated by post-translational modifications. We acknowledge that this study was restricted to transcriptomic datasets and peripheral blood validation. Comprehensive phenomic validation would require the integration of proteomic, metabolomic, imaging, and clinical phenotypic data (46). Future studies incorporating multi-omics and longitudinal phenomic analyses are warranted to further advance the phenomics paradigm.

We conducted a bioinformatics analysis using data from the GEO database to investigate the underlying molecular mechanisms, key genes, and patterns of immune cell infiltration associated with hyperthyroidism and AF. Using two machine learning algorithms—LASSO and RF—we identified CXCL16 and TMEM127 as potential diagnostic biomarkers and therapeutic targets for both conditions, offering a foundation for future investigations and potential therapeutic developments.

## Data Availability

The original contributions presented in the study are included in the article/Supplementary Material, further inquiries can be directed to the corresponding author.
