# Supplementary material for: Combining WGCNA and machine learning to identify mechanisms and biomarkers of hyperthyroidism and atrial fibrillation
Source: Front Cardiovasc Med. 2025 Nov 20;12:1694255. doi: 10.3389/fcvm.2025.1694255 (PMC12675458; doi:10.3389/fcvm.2025.1694255)
Supplement: Supplementary file 4 [file Table4.docx]

**Table S4-Basic characteristics of patients**

| Characteristics | Control (n = 16) | Hyperthyroidism (n = 16) | Atrial fibrillation (n = 16) |
| --- | --- | --- | --- |
| Male (%) | 7（30.4%） | 6（26.1%） | 10（43.5%） |
| Age (year) | 48.13 ± 11.69 | 48.88 ± 13.11 | 55.56 ± 9.81 |

Data is presented as mean value ± standard deviation or number or percentage of patients.
