# Supplementary material for: Combining WGCNA and machine learning to identify mechanisms and biomarkers of hyperthyroidism and atrial fibrillation
Source: Front Cardiovasc Med. 2025 Nov 20;12:1694255. doi: 10.3389/fcvm.2025.1694255 (PMC12675458; doi:10.3389/fcvm.2025.1694255)
Supplement: Supplementary file 5 [file Table5.docx]

**Supplementary Table S5** **- Quantitative Real-time PCR Primer List**

| **Name** | **Species** | **Forward** | **Reverse** |
| --- | --- | --- | --- |
| **GAPDH** | **Human** | AAAAGCATCACCCGGAGGAGAA | AAGGAAATGAATGGGCAGCCG |
| **TMEM127** | **Human** | CTGGTTGCACATCCACGGA | CTGGGGATTCATGCAGAAATCT |
| **CXCL16** | **Human** | CCCGCCATCGGTTCAGTTC | CCCCGAGTAAGCATGTCCAC |
